# Exploring time- and frequency- dependent functional connectivity and brain networks during deception with single-trial event-related potentials

**DOI:** 10.1038/srep37065

**Published:** 2016-11-11

**Authors:** Jun-feng Gao, Yong Yang, Wen-tao Huang, Pan Lin, Sheng Ge, Hong-mei Zheng, Ling-yun Gu, Hui Zhou, Chen-hong Li, Ni-ni Rao

**Affiliations:** 1 Key Laboratory of Cognitive Science of State Ethnic Affairs Commission and Laboratory of Membrane Ion Channels and Medicine, College of Biomedical Engineering, South-Central University for Nationalities, Wuhan, China; 2 Hubei Key Laboatory of Medical Information Analysis & Tumor Diagnosis and Treatment, Wuhan, China; 3 Key Laboratory for NeuroInformation of Ministry of Education, School of Life Science and Technology, University of Electronic Science and Technology of China, Chengdu, China; 4 School of Information Technology, Jiangxi University of Finance and Economics, Nanchang, China; 5 Key Laboratory of Oceanographic Big Data Mining & Application of Zhejiang Province, Department of Physics, Zhejiang Ocean University, Zhoushan, China; 6 Key Laboratory of Child Development and Learning Science of Ministry of Education, Research Center for Learning Science, Southeast University, Nanjing, Jiangsu, China; 7 Key Laboratory of Biomedical Information Engineering of Education Ministry, Institute of Biomedical Engineering, Xi’an Jiaotong University, Xi’an, China; 8 Department of Breast Surgery, Hubei Cancer Hospital, Wuhan, China

## Abstract

To better characterize the cognitive processes and mechanisms that are associated with deception, wavelet coherence was employed to evaluate functional connectivity between different brain regions. Two groups of subjects were evaluated for this purpose: 32 participants were required to either tell the truth or to lie when facing certain stimuli, and their electroencephalogram signals on 12 electrodes were recorded. The experimental results revealed that deceptive responses elicited greater connectivity strength than truthful responses, particularly in the *θ* band on specific electrode pairs primarily involving connections between the prefrontal/frontal and central regions and between the prefrontal/frontal and left parietal regions. These results indicate that these brain regions play an important role in executing lying responses. Additionally, three time- and frequency-dependent functional connectivity networks were proposed to thoroughly reflect the functional coupling of brain regions that occurs during lying. Furthermore, the wavelet coherence values for the connections shown in the networks were extracted as features for support vector machine training. High classification accuracy suggested that the proposed network effectively characterized differences in functional connectivity between the two groups of subjects over a specific time-frequency area and hence could be a sensitive measurement for identifying deception.

Lying is a ubiquitous social phenomenon, and lie detection (LD) has important legal, moral and clinical implications^1^. Over the past two decades, LD methods based on central nervous system activity, such as functional magnetic resonance imaging (fMRI) and event-related potential (ERP), have been vigorously developed^2^. The P300 wave has been extensively investigated and successfully used for LD for over 20 years^3^. Conventionally, ERPs are extracted by averaging across an ensemble of trials^4^,^5^. This ensemble-averaging approach assumes that ERPs are time-locked to stimuli and can be superimposed on independent, stationary, stochastic electroencephalogram (EEG) signals. However, ERPs are time-varying signals^6^,^7^, and the latency and amplitude of P300 waves vary by trial^8^. Furthermore, repetitive stimulus exposure increases the probability of producing fatigue and causing subjects to adopt countermeasures^9^,^10^. To overcome the above shortcomings, a spectrum calculated from the Fourier transformation was applied in a trial investigating lying^6^,^10^,^11^,^12^.

When using Fourier analysis, temporal information is lost by definition, and therefore the spectrum is assumed to remain constant. Notably, wavelet transforms (WTs)^7^ are highly suitable for ERP analysis at the single-trial level^13^. Indeed, a collection of previous LD studies have applied WTs during LD to improve detection accuracy^9^,^11^,^12^,^14^,^15^.

Understanding brain function requires not only gathering information from active brain regions but also studying functional interactions among neural assemblies distributed across different brain regions. Brain functional connectivity is a widely used measurement that characterizes correlations among the activities of different neural assemblies^16^,^17^. There are three typical calculation methods used in the field of linear function connectivity: cross-correlation, coherence and wavelet coherence (WC)^18^,^19^. When addressing non-stationary signals, it is recommended that time-frequency analysis be used, where the spectrum is estimated as a function of time^19^. WC is a technique that calculates the coherence between two time-frequency spectrums using WTs of two signals^20^.

High temporal resolution is one advantage that EEG has over fMRI. Unlike EEG-based analysis methods, fMRI cannot provide an accurate time period of synchronization because of its insufficient temporal resolution. Furthermore, most studies using fMRI-based LD methods have attempted to identify activated brain regions associated with a deceptive response^21^,^22^,^23^,^24^; however, these investigations have not performed in-depth analyses of functional connectivity across brain regions and therefore have not answered questions regarding how activated brain regions cooperate and synchronize (e.g., they have not identified connectivity strengths). In the present study, we hypothesized that a functional connectivity network is formed during deception that involves communication between specific brain-scalp regions over a specific time-frequency domain.

This study was conducted with the following aims: 1) to research LD from the perspective of functional connectivity in the brain and to test the feasibility of applying WC in single trials for analyzing synchronization between different brain sites; 2) to verify the hypothesis that significant differences exist in WC values between specific brain sites for specific time-frequency areas; 3) to propose a lying-associated functional connectivity network (LFCN) to characterize the pattern of coupling that forms between different brain-scalp regions during lying; and 4) to use this network to propose a classification model for identifying guilty and innocent subjects.

## Materials and Methods

### Subjects and EEG recording

This study was approved by the Psychology Research Ethical Committee of the College of Biomedical Engineering in South-Central University for Nationalities and was conducted in accordance with the most recent version of the Helsinki Declaration. Thirty-two healthy subjects (20 males) ranging in age from 20 to 24 years (mean, 22.6 years) with no history of neurological or psychiatric disease were recruited from the university. After a complete description of the study was provided to the participants, written informed consent was obtained. A 32-channel Synamps amplifier (NeuroScan, version 4.3, Charlotte, NC, USA) was used for EEG recording. Twelve standard scalp electrodes, including Fp1, Fp2, F3, Fz, F4, C3, Cz, C4, P3, Pz, P4 and Oz, were placed in accordance with the International 10–20 system^13^. The common average was used as a reference to the ground at Fpz, and the electrode impedances were maintained below 2 kΩ. EEG recordings were performed within an electromagnetic-shielded room. The EEG data were digitized at 500 Hz with a 0.3 to 100 Hz bandpass filter. Vertical and horizontal EOG signals were recorded to reject eye movement using an auto-regressive model applied in the Neuroscan software system. The EOG artifact removal criterion was set as ±75 μν in the Neuroscan software system.

### Experimental procedure

The guilty knowledge test (GKT)^15^ and the three-stimulus protocol^12^ were used in this study. The probe (P) stimuli consisted of images (or sounds) that were related to criminal acts, such as a weapon at the scene of a crime. The guilty individuals were familiar with these stimuli, whereas the innocent ones were not. The target (T) stimuli were known by all subjects, and these stimuli were not related to criminal acts. The irrelevant (I) stimuli were not known by all subjects and were also not related to criminal acts.

The participants were randomly divided into a guilty group and an innocent (i.e., control) group. There were no significant differences between the two groups in terms of age, gender or handedness. Six different jewels were prepared, and their pictures served as stimuli during the experiment. Each subject was trained and then asked to perform a mock crime scenario. A safe containing one (for innocent subjects) or two (for guilty subjects) jewels was given to each subject. The subjects were instructed to open the safe and memorize the details of the objects contained within. For the innocent subjects, the object in the safe was the T stimulus. Next, one of the remaining five images was randomly appointed to be the P stimulus. The other four images served as the I stimuli. In contrast, the guilty subjects were asked to steal one jewel that would serve as the P stimulus during the detection task, while the jewel remaining in the safe served as the T stimulus, and the remaining four pictures served as the I stimuli. When the subjects stole the jewels, all researchers were asked to stay outside of the room.

After being trained in the mock crime scenario, each subject was asked to sit on a chair that was placed approximately 70 cm from a computer screen, on which the stimulus pictures were presented pseudo-randomly without immediate repetition. Each stimulus remained on the screen for 500 ms, and the inter-stimulus interval varied randomly between 1.6 and 1.8 s. All subjects were instructed to respond to each stimulus as quickly as possible by pressing buttons. The innocent group responded honestly to all stimuli, whereas the guilty group was instructed to press the “No” button when faced with the P stimulus in an attempt to hide the stealing act. Each session lasted approximately 5 minutes, followed by a 3-minute rest period. In each session, each stimulus was randomly repeated 30 times, resulting in approximately 30 P, 30 T and 120 I responses. Each subject underwent 4 sessions. Behavioral data (response time and type) were recorded and embedded into the EEG signals using NeuroScan Stim2 software to enable correct segmentation of the EEG data. The guilty group was told that the task bonus would be 100 RMB if they successfully concealed the identity of the P stimuli during the detection task. One guilty and one innocent subject each had a clicking error rate greater than 5% and were initially excluded.

### Data preprocessing and time-domain analysis

Each continuous raw EEG recording was first segmented into epochs of −0.3 s to +1.3 s relative to the stimuli onset using the EEGLAB toolbox^25^. The response time was allowed to range from 0 s to 700 ms relative to the stimuli onset. Epochs with clicking error or too long of a response time (>700 ms) were rejected by visual inspection. Finally, twenty-six P trials were obtained in each session for each subject, resulting in a total of 1560 trials (26 trials × 4 sessions × 15 subjects) for each group. These trials were used for subsequent analysis. All epochs were then baseline-corrected based on the pre-stimulus interval. The grand averages of the trials within each subject were first calculated according to stimulus type and then checked by an independent neurophysiological expert to assess the general effect of the experiment. During the checking, if no P300 component was found in the target responses for a subject, all the experimental data from that subject were excluded on the basis that he/she did not concentrate on the experimental task.

### WC analysis

**Figure 1:**
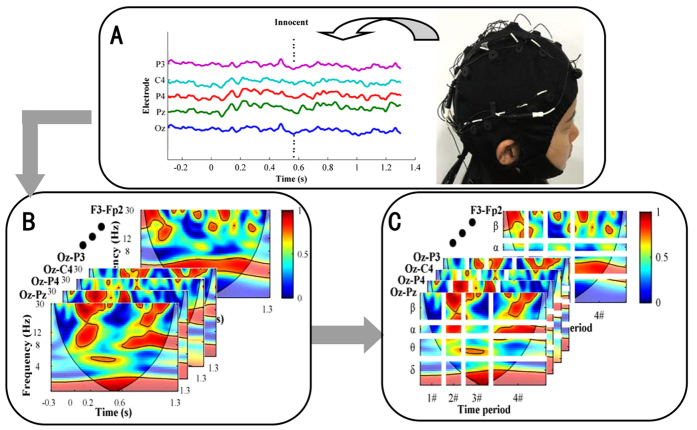
Diagram depicting WC analysis. (**A**) An exemplary trial from an innocent subject. (**B**) WC maps were calculated for 66 electrode pairs from the trial shown in Fig. 1A. (**C**) Divisional WC maps calculated from the WC maps. 1#, 2#, 3# and 4# in the time axis represent the pre-stimulus, pre-response, response and post-response periods, respectively. *δ* band: 0–4 Hz, *θ* band: 4–8 Hz, *α* band: 8–13 Hz, and *β* band: 13–30 Hz.

Figure 1 shows the procedure used for WC analysis. The WC algorithm^26^ was applied between any two electrodes within each trial (Fig. 1A), resulting in the generation of 66 (12 × 11/2 possible electrode pairs) WC maps (Fig. 1B) for each trial. This study was conducted with a particular interest in comparing WC among 4 specific frequency bands. Hence, similarly to past studies^27^,^28^, each WC map was divided into 16 time-frequency areas (4 bands: *δ*(0.1–4 Hz), *θ*(4–8 Hz), *α*(8–13 Hz) and *β* (13–30 Hz); 4 time periods: pre-stimulus, pre-processing, P300-processing and post-response), and the WC values within each divided area in each map were averaged. As a result, each original WC map was transformed into a divisional WC map (Fig. 1C).

### Statistical analysis

Statistical analyses were performed using SPSS Statistics v17.0 (SPSS Inc, Chicago, USA). Alpha significance was set at 0.05. To compare the WC values on the divisional maps between the two groups, a statistical group comparison was conducted for each electrode pair using an *independent samples t-test* over each time-frequency area with the subject type as the grouping variable (*degree of freedom* (*df*) = 3118). Additionally, as multiple comparisons were used, all *p* values were corrected by false discovery rate (FDR < 0.05) to control for false positives. Accordingly, a 12 × 12 statistical matrix was obtained for each time-frequency area, where the diagonal arrays were zero. The element value in the matrix was used to indicate whether there was a significant difference in a given electrode pair between the two groups—i.e., the value equaled 1 when *p*_*FDR−corrected*_ < 0.05 or 0 when *p*_*FDR−corrected*_ < 0.05.

Based on the statistical matrixes in the 1# and 3# periods for each frequency band, we selected only the electrode pairs for which a significant difference in WC values was found between the two groups during the 3# period (*p*_*FDR*−*corrected*_ < 0.05) and no significant difference was found during the 1# period (*p*_*FDR−corrected*_ ≥ 0.05). Hence, the following algorithm was separately used for each frequency band:

1. Let matrix **M1** and **M3** represent the statistical matrix during the 1# and 3# periods, respectively.
2. Let 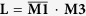, where the symbol—denotes logical NOT operation in each element in matrix **M1** and the symbol • denotes logical AND operation on two corresponding elements of two matrixes.
3. Obtain all the electrode pairs corresponding to value 1 in statistical matrix **L**, each of which is hereafter referred to as a *selected lying connection*.

### LFCN construction and classification

Based on all the selected lying connections in each frequency band, a LFCN was constructed. In this network, the strength of each line is represented by the mean difference in WC value during the 3# period between the two groups on corresponding connections. Hence, four time- and frequency-dependent LFCNs were obtained: *δ*, *θ*, *α* and *β*.

A classification model was set up for each LFCN. In each model, two classes of WC values (guilty group vs. innocent group) were used for all the selected lying connections to construct feature vectors. Hence, for each model, the dimension of the feature vector was equal to the number of selected lying connections in the corresponding network. A support vector machine (SVM)^11^,^15^ with the radial basis kernel function (RBF) (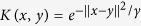) was selected as the classifier. A 10-fold cross-validation (CV) procedure^9^,^11^,^12^,^15^,^29^ was performed to form training and testing sets. For each fold, LIBSVM software was first used to tune classifier parameters with a searching grid of regularization parameter *C* and radial width *γ* for the kernel function (*C* = 2^−5^, 2^−4^, …, 2^4^, 2^5^; *γ* = 2^−5^, 2^−4^, …, 2^10^, 2^11^, 2^12^), which was then used during the training stage to train the classifier. The sensitivity and specificity from the training and testing phases were reported.

## Results

### Time-domain analysis

**Figure 2:**
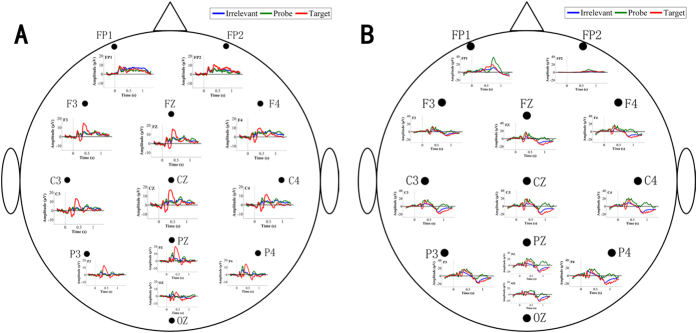
Grand-averaged P, T and I responses in (**A**) the innocent group and (**B**) the guilty group.

Figure 2 shows the grand-averaged P, T and I responses from the 12 electrodes in the two groups. First, we observed an obvious P300 component in the T response from both groups on some electrodes, such as Pz, P3, C3, C4 and Cz. In addition, there were no P300 components in the I responses of the two groups for any of the electrodes. Third, and most importantly, on some electrodes (e.g., Pz, P3, C3, C4 and Cz), a remarkable P300 component was found in the P responses of only the guilty group and not the innocent group. Furthermore, we found that ERPs existed from approximately 200 ms to 600 ms relative to the stimuli onset. Hence, we divided each trial into four time periods, as shown in Fig. 1C: 1# (−300–0 ms), 2# (0–250 ms), 3# (250–600 ms), and 4# (600–1300 ms). Combined with the above results, we selected the P responses for the following processing.

### WC analysis

**Figure 3:**
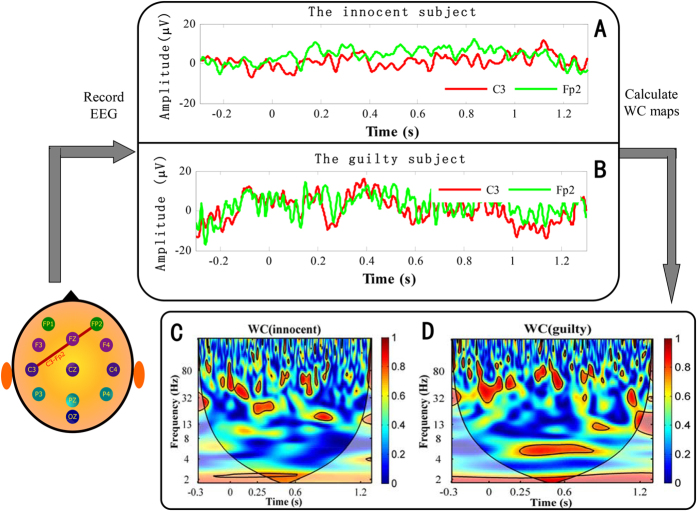
Comparison of four waveforms and their WC maps. (**A**) Two waveforms at C3 and Fp2 from an innocent subject. (**B**) Two waveforms at C3 and Fp2 from a guilty subject. (**C**) The WC map computed on the two signals shown in Fig. 3A. (**D**) The WC map computed on the two signals shown in Fig. 3B. Outside of the color cone, WC results cannot be given based on the computation rule of WC. For the significance of the thick black contour, see the report^26^,^57^.

First, the WC maps of one randomly selected pair of subjects were compared on the C3-Fp2 electrode pair. Figure 3 illustrates the results. The color bar in Fig. 3C,D reflects the strength of the WC values. The WC map between the two time series, shown in Fig. 3A, is displayed in Fig. 3C. Similarly, two time series from the guilty subject and his WC maps are shown in Fig. 3B,D. Comparing Fig. 3A,B, it is difficult to differentiate the trials of the two subjects. However, comparing Fig. 3C,D, a significant difference can be noted in the *θ* band for the period 350–600 ms. High coherence (indicated in red) can be observed in Fig. 3D over the time-frequency area mentioned above, whereas no such coherence exists in Fig. 3C over the same area.

**Figure 4:**
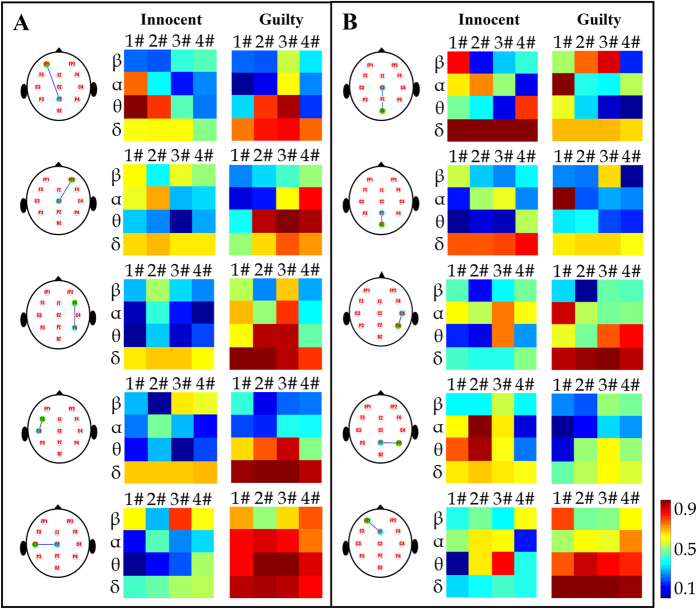
Comparison of averaged WC maps between one innocent subject and one guilty subject on (**A**) five electrode pairs with significantly greater WC values for the guilty subject than for the innocent subject in the *θ* band during the 3# period and on (**B**) five other electrode pairs, which show an unremarkable difference in the *θ* band during the 3# period.

Next, each subject’s divisional WC maps on each electrode pair were further averaged across trials, resulting in an averaged WC map for each electrode pair. For this experiment, one guilty and one innocent subject were randomly selected to show their averaged WC maps. Due to the limited amount of space, Fig. 4 illustrates the comparison of averaged WC values on 10 electrode pairs between the two selected subjects. In Fig. 4A, a highly significant difference is found in the *θ* band during the 3# period on these electrode pairs. However, with regard to the electrode pairs shown in Fig. 4B, there is no remarkable difference over the same area.

### Statistical analysis of WC values

**Figure 5:**
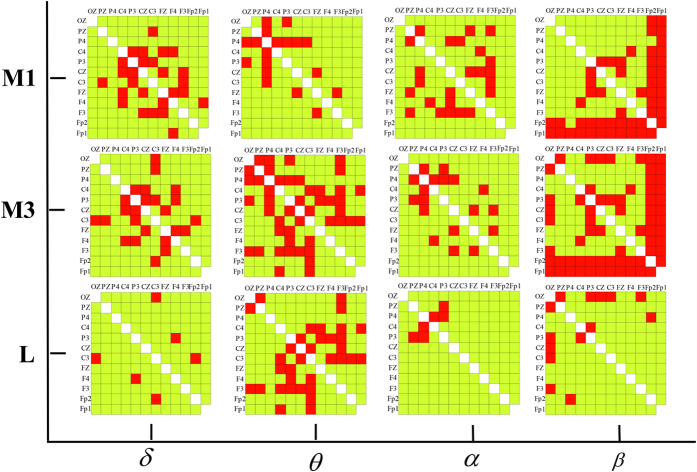
Statistical matrixes **M1** (the first row, the 1# time period), **M3** (the second row, the 3# time period) and **L** (the last row) for 4 frequency bands. In matrixes **M1** and **M3**, the red color denotes a significant difference (*p_FDR_*_−*corrected* _< 0.05, element value is equal to 1), and the yellow color denotes no significant difference (*p_FDR_*_−*corrected* _≥ 0.05, element value is equal to 0) for each corresponding pair between the two groups. In matrix **L**, the red color corresponds to an element value of 1, whereas the yellow color corresponds to an element value of 0.

Figure 5 shows statistical matrixes **M1**, **M3** and **L** for 4 bands. Note that in matrix **L**, for each frequency band, the red color denotes that significant differences were found only during the 3# period and not during the 1# period on those corresponding pairs, which are the *selected lying connection*s.

The last row in Fig. 5 shows that there are relatively more red blocks in the *θ* band than in the other three bands. That is, the statistical analysis of the WC values in the *θ* band revealed a significant group effect during the 3# period but simultaneously did not find an effect during the 1# period on most electrode pairs, such as Pz-Oz (*p*_*FDR*−*corrected*_ = 0.00007 during the 3# period, *p*_*FDR*−*corrected*_ = 0.084 during the 1# period, *df* = 3118), Pz-F3 (*p*_*FDR*−*corrected*_ = 0.0003 during the 3# period, *p*_*FDR*−*corrected*_ = 0.063 during the 1# period, *df* = 3118) and F3-Cz (*p*_*FDR*−*corrected*_ = 0.0001 during the 3# period, *p*_*FDR*−*corrected*_ = 0.256 during the 1# period, *df* = 3118). In contrast, in the *α* band, only 3 electrode pairs are marked in red, including P3-Pz (*p*_*FDR*−*corrected*_ = 0.0016 during the 3# period, *p*_*FDR*−*corrected*_ = 0.06 during the 1# period, *df* = 3118) and P3-P4 (*p*_*FDR*−*corrected*_ = 0.0007 during the 3# period, *p*_*FDR*−*corrected*_ = 0.142 during the 1# period, *df* = 3118). Similarly, only 3 connections in the *δ* band and 7 connections in the *β* band are marked in red.

**Figure 6:**
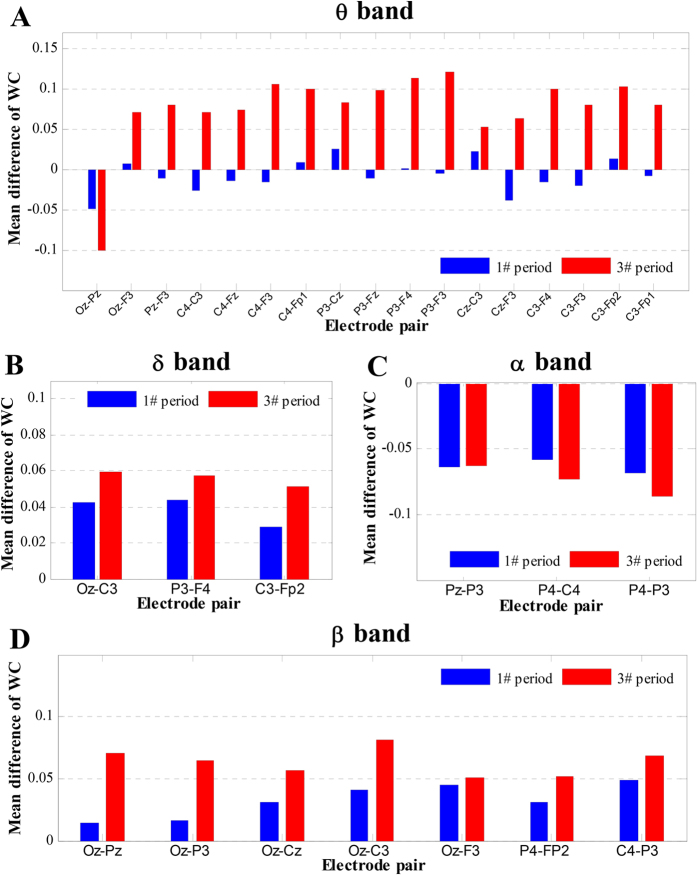
Group mean differences in WC values between the two groups of subjects, with a comparison between the 1# and 3# time periods for 4 frequency bands.

Furthermore, for each selected lying connection, the group mean of the WC values was first calculated for each period in each band. Then, the mean difference between the two groups (guilty minus innocent) was calculated. The mean differences corresponding to the 1# and 3# periods were defined as Δ1 and Δ3, respectively. The bars for Δ1 and Δ3 are shown in Fig. 6. Figure 6B,C show that all Δ1 differences are positive for the *δ* band, and all Δ3 differences are negative for the *α* band. Second, the differences between Δ1 and Δ3 are remarkable only for the electrode pairs Oz-C3 and C3-Fp2 for the *δ* band. However, Fig. 6A reveals a significantly higher mean difference in Δ3 than in Δ1 on all the electrode pairs. This finding indicates that, in the *θ* band, the functional connectivity of almost all the selected lying connections was very weak during the resting stage for the two groups, whereas the connectivity strength of the same connection significantly increased compared to the innocent subjects once a guilty subject presented a lying response. In Fig. 6D (*β* band), there is a remarkably greater mean difference in Δ3 than in Δ1 for almost all 6 electrode pairs (except Oz-F3). However, based on visual comparison, the differences between Δ3 and Δ1 on these 6 electrode pairs are smaller than the differences in almost all pairs in the *θ* band (expect Cz-C3).

**Figure 7:**
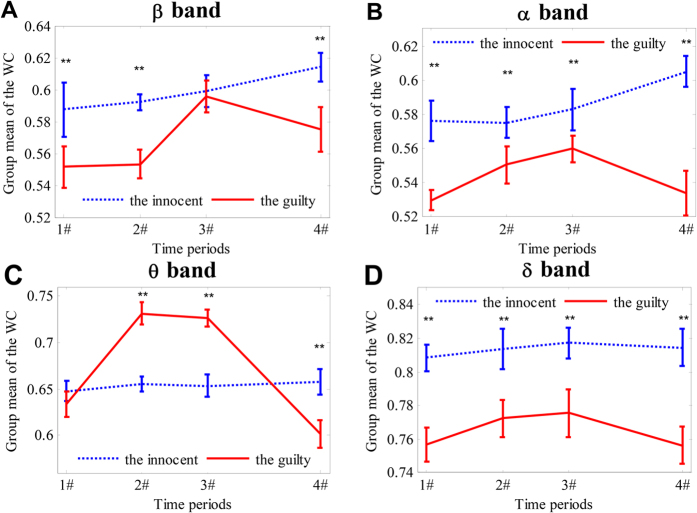
Changing courses in the group mean of the WC values on C4-Fz as a function of time period in the four frequency bands. (**A**) *β* band; (**B**) *α* band; (**C**) *θ* band; and (**D**) *δ* band. The values are given in the form of the mean ± SD. **Denotes *p* ≤ 0.0025 = 0.01/4 when statistically comparing the mean WC between the guilty group and the innocent group (Bonferroni correction was applied).

Finally, to display the changing courses of the WC values as a consequence of time, C4-Fz (one of the selected lying connections for the *θ* band) was chosen, and the WC values across the guilty subjects and the innocent subjects were averaged independently. Based on an *independent samples t-test* (Bonferroni correction was applied, *df* = 28), Fig. 7 illustrates the comparison of the group means (±standard deviation, SD) of the WC values as a function of time for the four frequency bands.

Figure 7C shows that the WC value for the guilty group increased significantly from 0.63 during the 1# period to 0.725 during the 3# period and finally decreased to a similar level to that of the pre-processing stage (0.60) during the 4# period. This finding suggests that the subject elicited higher functional connectivity between the right central brain region (C4) and the middle frontal region (Fz) during the stages of preparing and presenting a lying response than during the 1# period (i.e., the resting stage). In contrast, the WC value for the innocent group did not significantly increase or vary from the resting to the P300-processing stages, indicating that brain functional connectivity does not strengthen between these two regions when a subject is performing an honest action versus when they are at rest. In addition, the WC value for the guilty group was significantly higher than that for the innocent group during the 3# period (*p_FDR_*_−*corrected*_ < 0.0025 with Bonferroni correction, *df* = 28), demonstrating that the guilty group exhibited stronger brain connectivity than the innocent group while preparing and presenting a response to a stimulus. Correspondingly, the Δ3 between the two groups in the *θ* band is 0.08, whereas no value as large as this is found between the two groups in the other three frequency bands. Repeating the above analysis on the other selected lying connections in matrix **L** (see Fig. 5), we obtained similar results to those for electrode pair C4-Fz.

### LFCN

**Figure 8:**
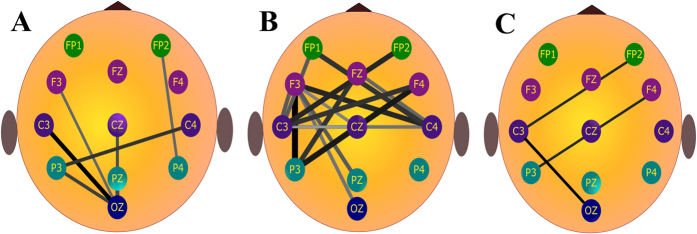
Lying functional connectivity networks for the (**A**)*β* band, (**B**) *θ* band and (**C**) *δ* band. The lines represent the between-region connectivity, and their thickness and width reflect the Δ3 value (gray and narrow: low Δ3; black and wide: high Δ3). All connections satisfy *p*_*FDR*−*corrected*_ ≤ 0.05.

Based on the above statistical results, three LFCNs were constructed, as illustrated in Fig. 8. In these networks, the thickness of the edge between any two electrodes reflects the Δ3 value for the corresponding connections. It is worth noting that the selected lying connection for which the Δ3 value was negative was deleted from the LFCN because we focused on the selected lying connection on which there existed a significantly greater WC value in the guilty group than in the innocent group. Hence, the *α* LFCN is not shown in Fig. 8.

Similar to a report by Lu *et al*.^30^, the 12 channels were grouped into 10 brain-scalp regions (see Table 1). Note that the frontal and prefrontal regions, represented by electrodes Fp1, Fp2, F3, Fz and F4, are hereafter referred to as a joint F region.

Based on these networks, the main results can be described as follows. For the *θ* LFCN (Fig. 8B), the central brain region, represented by electrodes C3, Cz and C4, has very close connectivity with the joint F region. In particular, the left central region (C3) connects with the whole joint F region except for Fz, and the right central region (C4) connects with the whole joint F region except for Fp2 and F4. Furthermore, regarding the left parietal region, P3 connects with the whole frontal region. Considering the connectivity strengths of all the connections, the 5 strongest connections can be listed from strongest to weakest as follows: 1) P3- F3 (Δ3 = 0.121), 2) P3- F4 (Δ3 = 0.113), 3) C4-F3 (Δ3 = 0.105), 4) C3-Fp2 (Δ3 = 0.103), and 5) C4-Fp1 (Δ3 = 0.100). These findings can be summarized as follows. The *θ* LFCN primarily encompasses the left and right central regions-joint F region and the left parietal region-frontal region, indicating that when a subject gives a lying response, there is a significant increase in the functional connectivity with the global network between the left parietal region and the frontal region and between the left and right central regions and the joint F region.

Based on the *β* LFCN (Fig. 8A), we see that among all 7 connections, the five strongest connections all involve the occipital region, represented by Oz in this study [e.g., Oz-C3 (Δ3 = 0.082) and Oz-P3 (Δ3 = 0.071)]. For the *δ* LFCN (Fig. 8C), there are only 3 weak connections, and the strongest connection is Oz-C3 (Δ3 = 0.059).

### Classification accuracy

Three classification models based on three LFCNs were constructed, as shown in Fig. 8. Based on these models, the dimensions of the feature vector for classification were 17 (in the *θ* LFCN), 7 (in the *β* LFCN) and 3 (in the *δ* LFCN). The classification results, expressed as the mean with SD, are shown in Table 2.

Table 2 shows that the model using the *θ* LFCN significantly outperformed the other two classifiers (e.g., *p* = 0.0003, *df* = 18, for the model using the *θ* LFCN vs. the model using the *β* LFCN for the sensitivity of the training set). For the model using the *θ* LFCN, the mean sensitivity and specificity in the training stage are 95.38% and 96.08%, respectively. Additionally, when using the *θ* LFCN, the predictive power is also very high, reaching 93.80% and 94.61% for the sensitivity and specificity, respectively. A balance test accuracy of 94.21% strongly suggests that the proposed classification model using the *θ* LFCN was highly effective for classifying the two types of subjects.

## Discussion

The significance of this investigation was the evaluation of the possibility of adopting EEG functional coupling for the purpose of LD. Current EEG-based LD methods are based primarily on single-electrode measurements. In contrast, most fMRI-based studies have focused only on the exploration of activated brain regions when a subject engages in a lie. The functional connectivity of neuroelectric activities in different brain regions during lying has not yet been thoroughly investigated. WC was utilized in this study as a new LD index to characterize EEG functional connectivity between different brain regions during deception. In particular, LFCNs based on different frequency bands were proposed in this study. To the best of our knowledge, this study is the first to generate a LFCN.

### Function and cooperation of activated brain regions in the LFCN

It is necessary to point out that our functional connectivity analysis was based on brain-scalp regions instead of on brain-cortical regions. The experimental results in this study indicate that when lying occurs, the prefrontal, frontal, parietal and central scalp regions were synchronously activated, going with the neuroelectric activity, particularly in the *θ* band. Notably, this finding is largely consistent with most previous fMRI/EEG studies in terms of the examination of activated brain-cortical or brain-scalp regions.

Over the past two decades, an increasing number of studies have indicated that the prefrontal and parietal cortical or scalp regions play an important role in the process of lying^1^,^2^,^9^,^11^,^12^,^24^,^31^. To date, many investigators have found that the P300 wave is usually the largest at Pz (the middle parietal scalp region) and the smallest at Fz (the middle frontal scalp region), taking intermediate values at Cz (the middle central scalp region)^5^,^12^,^29^. Accordingly, many EEG-based LD studies have acquired P300 waves on one of the scalp regions listed above^29^, which strongly suggests the important roles of the frontal, parietal and central scalp regions in LD. Despite the diversity of previous studies, one of the most consistent findings is that, compared with honest responses, lying engenders greater activity within and hence activates the joint F region (including the corresponding cortical regions), which plays a crucial role in processing the lying. Based on the *θ* LFCN presented in this study, we also found that the joint F region has an important role in lying.

Furthermore, most existing studies have indicated that the parietal regions are related to the execution of deception^32^. Johnson-Frey *et al*.^33^ reported that the left parietal region is a critical node for the planning of skilled movement; this finding agrees with our current results showing that P3 had a stronger and more connected relationship with the whole frontal region than Pz or P4 (see Fig. 8B). Additionally, based on the *θ* LFCN, the two strongest connectivities among all connections were found at P3- F3 (Δ3 = 0.121) and P3- F4 (Δ3 = 0.113). It should be stressed that the present study specifically focused on comprehensively and deeply interpreting the cooperative relationship between the abovementioned activated brain regions when lying occurs because the connectivity strength in the LFCN represents the difference in the functional connectivity between the two groups of subjects as opposed to the strength of the functional connectivity in only the guilty group.

Most existing fMRI-based LD studies claim to have analyzed neural correlations^32^. However, previous studies have only correctly identified activated brain regions when lying occurs and thus can be considered to have analyzed functional segregation^34^. In contrast, with the analysis of functional connectivity, our investigation indicated that three brain scalp regions—the prefrontal/frontal, central and parietal regions—were synchronously activated with different connectivity strengths when lying occurred and that these regions worked through a cooperative pattern of neural activity that demonstrated the obvious feature of low frequency.

### Time-frequency analysis of functional connectivity

Considering neuroimaging methodologies, fMRI, with a temporal resolution of approximately 1 s, is limited to modeling hemodynamic evoked responses. Poor temporal resolution makes it difficult to resolve dynamic changes in functional connectivity over a short period of time^30^. In contrast, EEG has high temporal resolution (<1 ms) and is therefore more optimal for the calculation of functional connectivity. In the present analysis, the synchronic time period for the neuroelectric activity in the proposed LFCN was limited to within only 350 ms, which is more accurate than the period analyzed by fMRI-based LD studies. This accuracy greatly contributes to an in-depth understanding of the dynamic processing that occurs during deception. The statistical results associated with the LFCNs proposed in this study demonstrate the advantages of our study over fMRI investigations.

The present study also has several advantages over most existing EEG-based LD studies. First, only a simple time or frequency analysis is used for most current LD methods. In contrast, time-frequency analysis can capture the overall features of non-stationary EEG signals. From this perspective, WC has merits over traditional time or frequency analysis due to the inherent advantages of wavelet analysis. Our experiment showed that the most significant difference between lying and telling the truth was found in the *θ* band during the 3# time period. Second, our study fills a gap between investigations based on a single electrode and studies involving multiple brain regions. In previous investigations, the measurements or features used to test lying were usually calculated and extracted from a single electrode^29^. In contrast, combining more electrodes or brain regions, such as in the present study, could make it easier to identify more valuable information, particularly when a certain task is accomplished via the cooperation of multiple brain sites. Accordingly, analysis of functional connectivity, such as correlation or coherence, can fully unearth key information from multiple brain sites. LD methods that use a single electrode are unable to accurately detect the wide distribution of neuroelectric activities that are present in lying responses^18^. The experimental results in the present study provide greater insight into the cooperative working patterns and neural connectivities that form between different brain regions during the production of lying responses.

### LD study using a single trial

Most current LD studies have required a number of stimuli to generate a complete identification result for one subject^4^,^5^. For example, the bootstrapped amplitude difference and the bootstrapped correlation difference^15^,^29^,^35^ methods cannot give an identification result until all stimuli are presented to the subject. However, the method proposed in the present study is based on the level of single trials; therefore, it is highly flexible for both testers and subjects^15^,^29^ and can greatly decrease subject fatigue and the risk of countermeasures. Second, compared with conventional ERP analysis^4^,^5^ and event-related coherence studies^36^, single-trial analysis investigating the distribution of coherence values for repeated stimuli^37^ could provide deeper insight into the neural mechanisms that underlie deception. For example, one could catch the time course associated with changes in functional coupling and then evaluate changes in neural mechanisms in the brain across trials. Third, in the present study, we emphasized that discrimination between lying and truth-telling should be achieved at the level of single trials to translate a theoretical study into a practical application^9^,^11^,^12^,^29^ instead of at the level of group analysis, which is favorable for LD and might also allow the possibility and increase the feasibility of implementing a real-time system for LD.

### Classification using the LFCN

It should be stressed that one of the most important purposes of LD is to distinguish guilty from innocent subjects. However, only a few studies using fMRI have proposed a classification method while simultaneously providing its sensitivity and specificity. In the present study, a machine learning-based classification method was presented to separate lies from truthful responses using WC-based features and a SVM. High classification accuracies, including high sensitivity and specificity, were obtained for the training and testing datasets, strongly supporting the view that it is reasonable and feasible to utilize the WC method in EEG to detect deceptive responses and hence to distinguish guilty from innocent subjects.

### Limitations

Interactions between different brain regions can be analyzed by bidirectional and unidirectional coupling^18^. The former can be assessed by functional connectivity, whereas the latter reflects a causal interaction between the proactor (the initiating source) and the reactor (the driven object). Obviously, neither previous LD work nor our current investigation can solve the latter coupling question. Hence, unidirectional coupling should be further assessed in future studies, which should increase understanding of the neural dynamics that are associated with the lying process. Various methods, such as a directed transfer function^38^, can then be applied to investigate the causal interaction mentioned above^18^,^39^.

Moreover, source domain connectivity has more reliable physiological interpretations when using EEG to analyze neural functional connectivity, for which the so-called inverse problem must be solved and the volume conductor effect should be considered^40^. Two major methods could be applied in this regard. A major solution to this problem is to compute the functional coupling between equivalent intracranial current dipoles. An inverse problem is an ill-posed problem^41^, and the optimal solution depends on many constraints and assumptions regarding information about dipoles, such as their moments, positions, magnitudes and orientations^42^,^43^. Additionally, a number of possible model configurations that fit well with the spatial patterns of scalp EEG potentials are needed^44^. It is obvious that many of the above factors will affect the accuracy with which a source can be localized^42^,^43^,^45^,^46^. Furthermore, theoretically, only an infinite number of recording electrodes could obtain the unique location of each of the responsible sources^47^. The second method consists of computing the coupling between brain regions of interest^48^,^49^, which still involves considerable subjective procession and a priori knowledge regarding the locations of neural current generators underlying scalp EEG recordings^44^. In sum, the localized sources within the brain are based on some a priori assumptions and knowledge^50^, so the locations are non-unique, and the connectivity analysis is still unreliable^42^,^45^. In the present study, a common average reference was first used to assure that WCs were independent in the reference of the EEG recording^30^,^38^. Moreover, we recorded EEG signals from 12 electrodes instead of 64 or more channels to increase the distance between the neighboring channels to the greatest degree possible, which could alleviate the negative effect of the volume conduction to some extent^38^,^51^. Kaminski and Blinowska noted that the spread of electrical activity becomes smeared by volume conduction, and they obtained clear and reproducible results in their study^51^. Similarly to many recent reports using EEG to assess brain functional connectivity^18^,^30^,^38^,^45^,^52^,^53^,^54^,^55^,^56^, we also assessed functional connectivity patterns in scalp regions instead of in the source domain. We emphasize that both the experimental results and the analyses in the present study were restricted to brain-scalp regions. Even so, as discussed previously, the brain-scalp regions that were identified as becoming activated during lying were largely consistent with the activated cortical regions identified in most previous fMRI studies. This concordance demonstrates that the results in the present study were hardly affected by volume conduction and are therefore credible. Evaluating the functional connectivity patterns that form during lying in the source domain is beyond the scope of this study, although we may investigate these patterns in future studies.

Finally, notwithstanding the good temporal resolution of EEG, our proposed approach also has the shortcoming of poor spatial precision. A growing number of studies have come to recognize the great advantages of employing both neuroimaging and neurophysiological methodologies. For example, a study by Sun *et al*.^23^ suggested that combining EEG and fMRI could provide insights into both the spatial and temporal features that are associated with the neural process of deception. Hence, enhancing the spatiotemporal accuracy of investigations into the neural process of lying is a future research goal that could be achieved using the multimodal fusion approaches mentioned above.

## Additional Information

**How to cite this article**: Gao, J.- *et al*. Exploring time- and frequency- dependent functional connectivity and brain networks during deception with single-trial event-related potentials. *Sci. Rep*. **6**, 37065; doi: 10.1038/srep37065 (2016).

**Publisher’s note:** Springer Nature remains neutral with regard to jurisdictional claims in published maps and institutional affiliations.

## Figures and Tables

**Table 1:** The divided scalp regions and their channels.

Scalp regions	Channels
Frontal region	F3, Fz, F4
Prefrontal region	Fp1, Fp2
Joint F region	Fp1, Fp2, F3, Fz, F4
Left central region	C3
Right central region	C4
Middle central region	Cz
Left parietal region	P3
Right parietal region	P4
Middle parietal region	Pz
Occpital region	Oz

**Table 2:** Classification accuracies (mean ± SD) using three different LFCNs.

Classifier models	Classification accuracies (%)			
	Training		Testing	
	*Sensitivity*	*Specificity*	*Sensitivity*	*Specificity*
Model using the *δ* LFCN	80.23 ± 3.42^▲^	81.16 ± 3.12^▲^	79.27 ± 3.01^▲^	78.98 ± 3.09^▲^
Model using the *β* LFCN	83.25 ± 2.77^*^	83.51 ± 2.00^*^	82.35 ± 2.45^*^	82.88 ± 2.57^*^
Model using the *θ* LFCN	95.38 ± 3.14	96.08 ± 2.72	93.80 ± 2.85	94.61 ± 2.74

“▲” denotes *p* < 0.01 for the statistical comparison between the model using the *θ* LFCN and the model using the *δ* LFCN; “*” denotes *p* < 0.01 for the statistical comparison between the model using the *θ* LFCN and the model using the *β* LFCN (*df* = 18).
